# Temporal changes in parameters associated with tear film stability after instillation of long-acting diquafosol ophthalmic solution in soft contact lens wearers

**DOI:** 10.1007/s10384-025-01161-y

**Published:** 2025-02-12

**Authors:** Takashi Itokawa, Takashi Suzuki, Hiroko Iwashita, Yukinobu Okajima, Koji Kakisu, Yuichi Hori

**Affiliations:** 1 Department of Ophthalmology, Toho University Faculty of Medicine, 6-11-1, Omori-Nishi, Ota-ku, Tokyo, 143-8541 Japan; 2 Ishizuchi Eye Clinic, Niihama, Ehime Japan; 3 Tsunashima Eye Clinic, Yokohama, Kanagawa Japan

**Keywords:** Tear meniscus height, Non-invasive tear break-up time, Long-acting diquafosol ophthalmic solution, Soft contact lens, Ocular surface temperature

## Abstract

**Purpose:**

To investigate temporal changes in parameters associated with tear film stability after instillation of long-acting 3% diquafosol ophthalmic solution (DQS LX), which contains polyvinylpyrrolidone.

**Study Design:**

Prospective crossover study design.

**Methods:**

We enrolled 23 eyes of 23 soft contact lens (SCL) wearers (aged 25.3 ± 4.4 years). One-day disposable silicone hydrogel lenses (narafilcon A) were used in this study. DQS LX was instilled without a SCL on the first day. On the other two days, subjects received DQS or DQS LX at 7 h after wearing SCLs. Tear meniscus height (TMH), non-invasive tear break-up time (NIBUT) and ocular surface temperature (OST) were measured before and at 5, 15, 30, 45, 60, 80 and 120 min after instillation.

**Results:**

When not wearing SCLs, DQS LX instillation significantly improved TMH for up to 120 min and NIBUT for up to 80 min. When wearing SCLs, DQS and DQS LX instillation significantly increased TMH for up to 45 and 80 min, and NIBUT for up to 15 and 30 min. Compared to DQS, DQS LX administration resulted in a significantly higher TMH at 5, 60 and 80 min, as well as a significantly prolonged NIBUT at 5 and 60 min. OST with and without SCLs also varied depending on the changes in the parameters associated with tear film stability.

**Conclusion:**

Compared to DQS, when wearing SCLs, DQS LX was found to increase the amount of tear fluid and improve tear film stability for longer periods.

## Introduction

Almost 50% of soft contact lens (SCL) wearers perceive dryness and discomfort towards the end of the day [1]. A SCL divides the tear film into pre- and post-SCL portions, with the pre-SCL tear film becoming thin and unstable after SCL insertion [2]. The instability of the tear film has an effect on the ocular surface and causes subjective symptoms due to increasing friction between the SCL and the eyelid or the bulbar conjunctiva, which can lead to a decrease in the function of the meibomian glands [3] and goblet cells [4], and an increase in the lid-wiper epitheliopathy [5]. Parameters that reflect the tear film stability include tear meniscus height (TMH) and tear break-up time (BUT). It is reported that TMH is correlated with BUT, which indicates that a lower TMH is associated with worse tear film stability and dryness symptoms [6–8].

In order to improve tear film stability, increases in the wetness of the SCL material [9], or the addition of topical ophthalmic solutions [10] are required. A topical ophthalmic solution, such as hyaluronic acid (HA), is used to reduce the corneal and conjunctival staining and improve subjective symptoms [10, 11]. In addition, 3% diquafosol tetrasodium ophthalmic solution (3% Diquas (DQS); Santen Pharmaceutical Co.) for the treatment of dry eye was launched in 2010 as a way to increase the amount of tear fluid over a long period of time, with DQS also administered to SCL wearers complaining of dryness [12, 13]. DQS is a P2Y_2_ purinergic receptor agonist. These receptors are present in the conjunctival epithelial cells, goblet cells and adipocytes and ductal epithelial cells in the meibomian glands [14]. When the P2Y_2_ receptor is activated, it promotes the secretion of aqueous fluid and mucin contents in tear fluids [15]. Some researchers report that there was a significant increase in the amount of tear fluid for up to 30 min after the use of DQS as compared to artificial tears [15, 16]. At the same time, the tear film stability was significantly improved for up to 20 min as compared to the use of artificial tears and HA [17]. Moreover, when wearing SCLs, the amount of tear fluid was significantly increased up to 60 min as compared to that observed when using artificial tears or before the instillation of DQS [18].

Although the recommended DQS administration is the application of eye drops six times a day, it is reported that the effectiveness of DQS may be insufficient when the specified number of eye drops is not adhered to [19]. Therefore, a long-acting diquafosol tetrasodium (3% Diquas LX (DQS LX); Santen Pharmaceutical) agent was launched in 2022. This drug contains a polymeric ingredient (polyvinylpyrrolidone; PVP) that has water retention properties in order to prolong the pharmacological action on the ocular surface, with DQS LX reducing the administration of the specified number of eye drops from six to three times a day [20, 21]. However, the temporal changes in the tear volume and tear film stability after instillation of DQS LX have yet to be definitively determined. It is also not known to what degree DQS LX improves these parameters as compared to DQS when subjects are wearing SCLs. The purpose of the present study was to investigate the temporal changes in parameters related to tear film stability after the instillation of DQS LX while wearing or not wearing SCLs. In addition, this study also investigated the differences between DQS LX and DQS over time while wearing SCLs.

## Subjects and methods

### Subjects

This study enrolled 23 eyes of 23 SCL wearers who habitually wore SCLs more than 5 days a week with an age between 20 and 39 years old (Table 1). We excluded SCL wearers who had self-reported dry eye symptoms when not wearing SCLs, who were taking medications known to affect the ocular surface, exhibited the presence of allergic keratoconjunctivitis, and had a history of ocular and systemic disease. This prospective crossover study, which adhered to the tenets of the Declaration of Helsinki, was approved by the Ethics Committee of the Faculty of Medicine, Toho University (No. A22075) and registered in the Japan Registry of Clinical Trials (jRCT) (Registry No. jRCT1031230239). All subjects provided written informed consent after a detailed explanation of the nature and possible consequences of the study.

**Table 1:** Characteristics of subjects

*N* = 23 eyes of 23 SCL wearers	
Age, years	25.3 ± 4.4
Sex, male: female	8:15
SCL wearing history, years	9.7 ± 5.2
SCL wear frequency, days/week	6.1 ± 1.0
TMH, mm	0.16 ± 0.09
FBUT, sec	5.2 ± 2.5
NIBUT, sec	5.5 ± 4.7
J-OSDI	20.1 ± 16.5
J-CLDEQ-8	15.2 ± 7.1

Data are shown as the mean ± standard deviation

*SCL* soft contact lens, *TMH* tear meniscus height, *NIBUT* non-invasive break-up time, *J-OSDI* Japanese version of ocular surface disease index, *J-CLDEQ-8* Japanese version of the 8-item contact lens dry eye questionnaire

## Assessment of ocular surface

TMH measurements were performed using Keratograph 5 M (Oculus) [22]. After focusing on the ocular surface, subjects were asked to close their eyes. Subsequently, immediately after subjects reopened their eyes, the meniscus image between the lower eyelid and ocular surface was captured and measured.

Non-invasive tear break-up time (NIBUT) was measured using interferometry (DR-1α, Kowa Co. Ltd.) [23]. Measurements were conducted after several blinks, with NIBUT defined as the time from the eye opening to the appearance of tear film break-up.

Ocular surface temperature (OST) was measured using an Ocular Surface Thermographer (TG1000, Tomey Corporation) [24]. After closing the eye for 5 s and then reopening the eye, measurements were immediately conducted within 1 s. The analysis area was set at 4 mm from the center of the cornea.

The fluorescein tear break-up time (FBUT) measurement was conducted using a fluorescein test strip (Fluores Ocular Examination Test Paper; Ayumi Pharmaceutical Co.) [25]. After wetting the strip with topical saline, it was shaken to remove excess water. Subsequently, the strip was softly touched to the lower lid margin, and then after several blinks, the FBUT was measured three times using a metronome. The average of three results was calculated as the representative value.

## Assessment of subjective symptoms

We used three types of questionnaires, the Japanese version of the Ocular Surface Disease Index (J-OSDI) [26], the Japanese version of the 8-item Contact Lens Dry Eye Questionnaire (J-CLDEQ-8) [27] and the visual analog scale (VAS) for dryness and foreign body sensation. We used the J-OSDI and J-CLDEQ-8 for the assessment of the symptoms in habitual SCL wearers both while wearing and while not wearing the SCLs. Changes in subjective symptoms, dryness and foreign body sensation between the before and after eye drops’ period, were evaluated using the VAS, which was assessed on a 0-to-100-point scale. The higher score for the J-OSDI, J-CLDEQ-8 and VAS indicated the presence of strong symptoms.

## Study protocol

Subjects visited the hospital on three different days. Time intervals of at least more than one day were scheduled between visits. On the day before the hospital visit, all subjects were asked to spend time without wearing SCLs. The study was performed between 15:00 and 20:00, with the temperature (25.0 ± 0.9 °C) and humidity (24.9 ± 7.5%) in the measurement room maintained at a constant level. Measurements were conducted only in the right eye.

At visit 1, subjects without SCLs were evaluated in the examination room. After 15 min of rest, subjective symptoms (J-OSDI, J-CLDEQ-8 and VAS), TMH, NIBUT, OST and FBUT were measured for the baseline values prior to the instillation of DQS LX. Subjects then received a single drop of DQS LX OU without any SCL, with measurements of the subjective symptoms (VAS), along with evaluations of the TMH, NIBUT and OST then conducted at 5, 15, 30, 45, 60, 80 and 120 min after the instillation.

Visit 2 and 3 were set as a crossover design during which subjects received DQS or DQS LX on different days while wearing SCLs. We used the narafilcon A daily disposable silicone hydrogel lens (1-day Acuvue TruEye; Johnson & Johnson) with the diopter unified at -0.5D. At visit 2, subjects were asked to wear the SCL for 7 h before visiting the hospital. After arriving at the hospital, subjects rested for 15 min, after which the subjective symptoms (VAS), along with the TMH, NIBUT and OST were measured. Subsequently, subjects received a single drop of DQS or DQS LX in both eyes, with the measurements after the eye drops then conducted at the same time points as for visit 1. At visit 3, the measurements were performed in the same manner as for visit 2, but with the eye drops changed to the other drug (DQS LX or DQS).

### Data analysis

Sample size was calculated based on the results of a previous study, which reports finding changes in the BUT of -0.38 and 1.28 s at 10 min after instillation of saline and DQS [17]; this indicated that at least 17 eyes were required for the present study design (α = 0.05, power 80%). The mean and standard deviation values were used for the statistical analysis of the data. The changes in the TMH, NIBUT and OST over 5, 15, 30, 45, 60, 80 and 120 min after the eye drops were based on the baseline values and analyzed by the Friedman test, followed by the Steel test as a multiple comparison test when there were significant differences. The Wilcoxon signed-rank test was used to compare the changes in parameters between the DQS and DQS LX. Spearman’s rank correlation coefficient was used to analyze the correlations between the subjective symptoms and parameters, i.e., TMH, NIBUT and OST. All analyses were performed using JMP Pro version 17 statistical analysis software (SAS Institute Inc.).

## Results

### Changes in ocular surface parameters when not wearing SCLs after DQS LX instillation

**Fig. 1:**
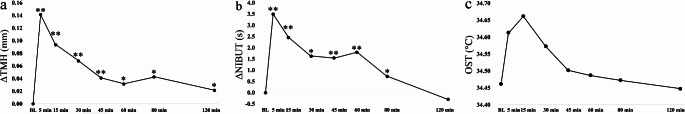
Graphs showing the temporal changes in TMH (**a**), NIBUT (**b**), and OST (**c**) after instillation of DQS LX when not wearing SCLs. Each data point represents the mean value. *P < 0.05, **<0.01 (in comparison with baseline, Steel test). *TMH* tear meniscus height, *NIBUT* non-invasive break-up time, *OST* ocular surface temperature, *DQS LX* long-acting 3% diquafosol ophthalmic solution, *SCL* soft contact lens

Changes in the TMH when not wearing SCLs after the administration of DQS LX at 5, 15, 30, 45, 60, 80 and 120 min were 0.14 ± 0.12, 0.09 ± 0.09, 0.07 ± 0.09, 0.04 ± 0.06, 0.03 ± 0.07, 0.04 ± 0.07 and 0.02 ± 0.05 mm, respectively. The changes in the TMH were significantly increased until 120 min as compared to the baseline values (*P* < 0.0001, *P* < 0.0001, *P* < 0.0001, *P* = 0.0002, *P* = 0.0119, *P* = 0.0376, *P* = 0.0314, Steel test, Fig. 1a).

Changes in the NIBUT were 3.5 ± 2.8, 2.5 ± 3.0, 1.6 ± 3.1, 1.5 ± 4.7, 1.8 ± 2.7, 0.7 ± 3.7 and − 0.3 ± 3.2 s, respectively. Differences in the NIBUT were significant until 80 min (*P* < 0.0001, *P* < 0.0001, *P* = 0.0119, *P* = 0.0070, *P* = 0.0054, *P* = 0.0377, Steel test, Fig. 1b).

While OST also varied depending on the changes in the tear film parameters, the changes were not significant (Fig. 1c).

## Changes in ocular surface parameters when wearing SCLs after DQS LX and DQS instillation

**Fig. 2:**
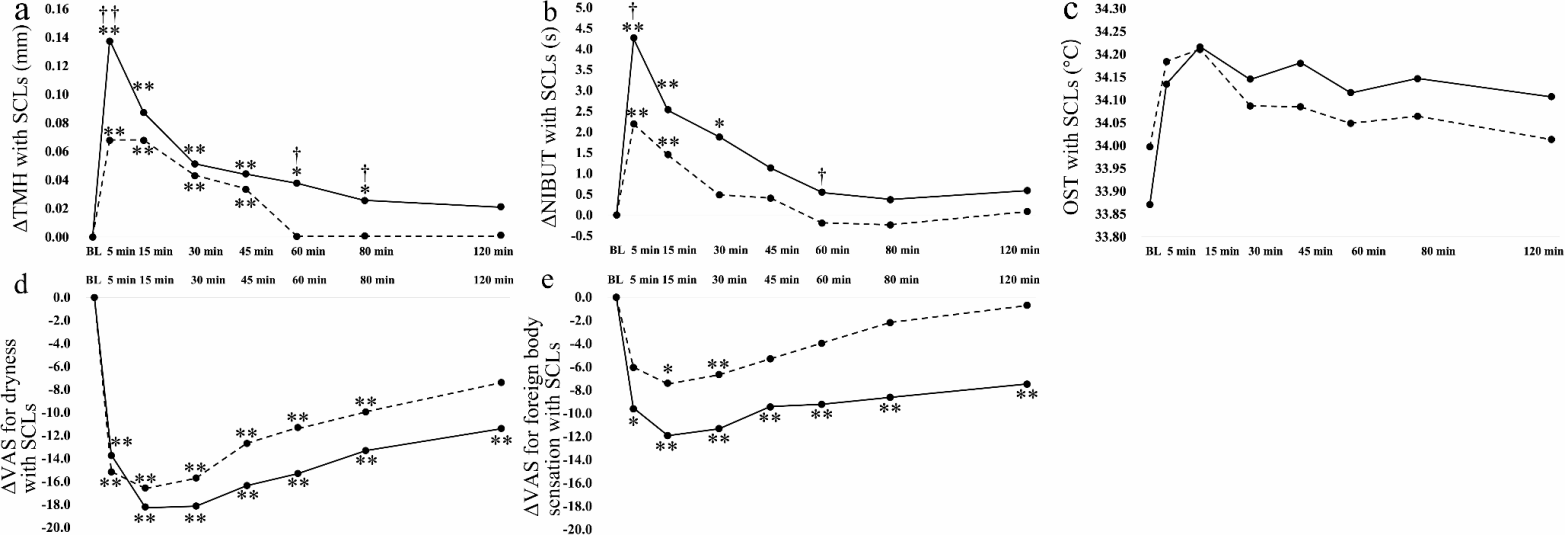
Graphs showing the temporal changes in TMH (**a**), NIBUT (**b**), OST (**c**), dryness (**d**), and foreign body sensation (**e**) after instillation of DQS and DQS LX when wearing SCLs. The solid line represents DQS LX instillation, while the dashed line represents DQS instillation. Each data point represents the mean value. *P < 0.05, **<0.01 (in comparison with baseline, Steel test). †*P* < 0.05, ††<0.01 (in comparison with DQS and DQS LX, Wilcoxon signed-rank test). *TMH* tear meniscus height, *NIBUT* non-invasive break-up time, *OST* ocular surface temperature, *DQS LX* long-acting 3% diquafosol ophthalmic solution, *SCL* soft contact lens

Changes in the TMH while wearing SCLs at 5, 15, 30, 45, 60, 80 and 120 min after instillation of DQS were 0.07 ± 0.05, 0.07 ± 0.08, 0.04 ± 0.08, 0.03 ± 0.07, 0.00 ± 0.04, 0.00 ± 0.04 and 0.00 ± 0.04 mm, respectively. The changes in the DQS LX were 0.14 ± 0.08, 0.09 ± 0.06, 0.05 ± 0.07, 0.04 ± 0.06, 0.04 ± 0.07, 0.03 ± 0.06 and 0.02 ± 0.06 mm, respectively. As compared to the baseline values, TMH values after instillation of DQS were significantly increased until 45 min (*P* < 0.0001, *P* < 0.0001, *P* = 0.0002, *P* = 0.0006, Fig. 2a), and after DQS LX, they were significantly increased until 80 min (*P* < 0.0001, *P* < 0.0001, *P* < 0.0001, *P* = 0.0004, *P* = 0.0314, *P* = 0.0375, Fig. 2a). As compared to DQS, DQS LX was significantly higher at 5, 60 and 80 min (*P* < 0.0001, *P* = 0.0234, *P* = 0.0417, Wilcoxon signed-rank test, Fig. 2a).

Changes in the NIBUT while wearing SCLs at 5, 15, 30, 45, 60, 80 and 120 min after instillation of DQS were 2.2 ± 2.2, 1.5 ± 3.1, 0.5 ± 1.6, 0.4 ± 1.5, -0.2 ± 1.0, -0.2 ± 2.0 and 0.1 ± 2.6 s, respectively. The values for DQS LX were 4.3 ± 4.1, 2.5 ± 3.9, 1.9 ± 3.4, 1.1 ± 3.3, 0.8 ± 2.1, 0.4 ± 3.4 and 0.6 ± 3.7 s, respectively. Compared to baseline, the NIBUT values after instillation of DQS were significantly prolonged up to 15 min (*P* < 0.0001, *P* = 0.0086, Fig. 2b), while for DQS LX they were significantly prolonged for up to 30 min (*P* < 0.0001, *P* = 0.0315, *P* = 0.0023, Fig. 2b). As compared to DQS, DQS LX values were significantly longer at 5 and 60 min (*P* = 0.0383, *P* = 0.0476 for both comparisons, Fig. 2b).

The OST while wearing SCLs after instillation of DQS and DQS LX varied depending on the changes found in the tear film parameters. However, the changes were not significant (Fig. 2c).

The changes in the dryness symptoms while wearing SCLs after instillation of DQS and DQS LX were significantly improved up until 80 (*P* = 0.0014, *P* = 0.0006, *P* < 0.0001, *P* = 0.0014, *P* = 0.0025, *P* = 0.0056, Fig. 2d) and 120 min (*P* = 0.0003, *P* < 0.0001, *P* < 0.0001, *P* < 0.0001, *P* < 0.0001, *P* = 0.0010, *P* = 0.0006, Fig. 2d), respectively. The changes in foreign body sensation symptoms while wearing SCLs were significantly improved at 15 and 30 min for DQS (*P* = 0.0142, *P* = 0.0068, Fig. 2e) and up until 120 min for DQS LX (*P* = 0.0309, *P* = 0.0032, *P* = 0.0032, *P* = 0.0068, *P* = 0.0032, *P* = 0.0032, *P* = 0.0068, Fig. 2e). However, these symptoms did not exhibit any significant differences between DQS and DQS LX at all of the measurement time points.

### Association between dryness symptoms and ocular surface parameters while wearing SCLs

**Fig. 3:**
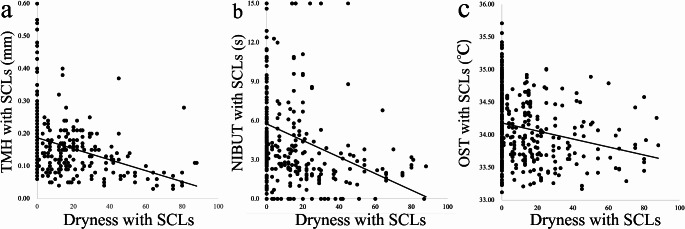
Graphs showing the correlation between dryness and **a** TMH (*r* = − 0.4078, *P* < 0.0001, Spearman’s rank correlation coefficient), **b** NIBUT (*r* = − 0.3457, *P* < 0.0001), and **c** OST (*r* = − 0.2543, *P* < 0.0001). T*TMH* tear meniscus height, *NIBUT* non-invasive break-up time, *OST* ocular surface temperature

Dryness was significantly associated with the TMH (*r* = -0.4078, *P* < 0.0001, Spearman’s rank correlation coefficient, Fig. 3a), NIBUT (*r* = -0.3457, *P* < 0.0001, Fig. 3b) and OST (*r* = -0.2543, *P* < 0.0001, Fig. 3c), indicating that when there was a higher dryness perception, there was lower TMH, NIBUT and OST.

## Discussion

In this study, after the instillation of DQS LX, the TMH when not wearing SCLs was significantly increased for up to 120 min, while the values for the NIBUT were significantly prolonged for up to 80 min. When wearing SCLs, although the TMH for DQS was significantly increased for up to 45 min and the NIBUT was significantly prolonged for up to 15 min, the TMH for DQS LX was significantly increased for up to 80 min, and for up to 30 min for NIBUT. The TMH for the DQS LX was significantly higher at 5, 60 and 80 min as compared to DQS, and the NIBUT for DQS LX was significantly longer at 5 and 60 min as compared to the DQS. The OST varied depending on the changes in the amount of tear fluid. Dryness and foreign body sensation symptoms when wearing SCLs were significantly improved for up to 120 min, and for 120 min after DQS LX, and for 80 min and 30 min after DQS. Results showed that the dryness was significantly correlated with the TMH, NIBUT and OST.

Previous studies show that topical instillation of DQS accelerates the aqueous fluid secretion from the conjunctival epithelial cells [28–30] and secreted mucin production from goblet cells [31–34] due to activation of the P2Y_2_ receptor. In studies of normal eyes that investigated the changes in tear fluid volume after instillation of DQS, the tear fluid was shown to be significantly increased for up to 30 min as compared to when using artificial tears [16, 35]. In SCL wearers, the tear fluid volume when wearing a SCL was significantly increased up until 30 to 60 min as compared to the use of artificial tears or before the instillation of DQS [18, 36]. In the present study, TMH when wearing SCLs after instillation of DQS was significantly increased for up to 45 min, which was consistent with the findings of past studies [18, 36].

The reason for the TMH and NIBUT values exhibiting significant differences at 5 min after the instillation of DQS LX compared to DQS was thought to be associated with the changes in the water retention [37] and viscosity [38] of the tear fluid due to PVP and the formation of electrostatic interactions between PVP, mucin and water [39, 40], as DQS LX uses the same concentration of diquafosol (3%) as that for DQS. The viscosity of the ophthalmic solution can have an impact on the tear fluid volume after instillation of the ophthalmic solution. In the HA ophthalmic solution, the greater the increase in the concentration of HA, the greater the increase in the viscosity and water retention [41, 42]. Carracedo et al. report that there was a significantly increased TMH when using 0.1% HA (viscosity (mPa); 4–8) and a saline solution (viscosity (mPa); 0.95–1.05) up until 1 min, up until 5 min when using 0.2% HA (viscosity; 10–20), and up until 10 min when using 0.3% HA (viscosity; 50–52) [43]. DQS (viscosity; 0.8–1.2) is reported to have the same viscosity as saline [35, 44]. For solutions containing PVP, the viscosity [45, 46] and water retention [47] properties increase as the molecular weight and concentration of PVP increases. However, because the water retention properties of PVP are not as high as those for HA, electrostatic interactions between PVP, mucin and water, as discussed below, may also be related to the changes in the TMH and NIBUT that are observed immediately after instillation of DQS LX.

The reason for the significantly different changes in the parameters associated with tear film stability as compared to DQS at 5 and 60 to 80 min was thought to be because the PVP in the DQS LX could form electrostatic interactions with mucin and water, thereby increasing the retention time of the tear fluid on the ocular surface [39, 40]. Baszkin et al. report that silicone hydrogel lens grafted by PVP adsorbed mucin much more than that observed for ungrafted lenses, with this effect becoming more significant when using higher concentrations of mucin dissolved in the solution [40]. Moreover, this phenomenon was observed to exhibit rapid changes during the first few minutes, and taking 10 to 18 h until the apparent plateau values. We previously evaluated subjects wearing hydrogel lenses with PVP and found that the NIBUT was significantly longer as compared to when using the same material without PVP [23]. In the present study, when applying the DQS LX containing PVP, the action of diquafosol promoted tear fluid and mucin production, which led to persistent long lasting stable tear film stability due to electrostatic interactions by the PVP, mucin and tear fluid. However, the SCL used in this study was the silicone hydrogel lenses containing PVP. Thus, results may vary depending on the type of material used (hydrogel, silicone hydrogel lenses or PVP based material), and whether or not it contains PVP. Further studies that evaluate this point will need to be conducted.

Dryness and discomfort were easily perceived after wearing SCLs, with the main cause of these symptoms thought to be due to the unstable prelens tear film stability [2]. Guillon et al. report that tear film stability in symptomatic SCL wearers was significantly worse than that observed in asymptomatic wearers [8]. Che et al. investigated the discomfort between 2 and 10 h after wearing SCLs, and based on the wearing time, found that the tear fluid volume was significantly decreased and the discomfort was worse. In addition, they also report finding an association between the tear fluid volume and the discomfort [7]. However, there are no reports on how a short-term improvement in the tear fluid volume after the instillation of eye drops is related to the perception of dryness. In the present study, we found there was an association between the dryness and the parameters related to tear film stability (TMH, NIBUT and OST). This result suggests the dryness improves immediately after an increase in the tear fluid volume. Moreover, the improvement in the dryness after the instillation of DQS LX was significantly longer as compared to after the instillation of DQS. These results suggest that DQS LX may be a more effective treatment for dryness when wearing SCLs.

There were some limitations in the present study. First, the present study included SCL wearers with and without CL discomfort, and it was not clarified whether DQS LX had the same effect on SCL wearers with and without CL discomfort. A future study will need to be conducted to determine the difference in the efficacy of DQS LX between SCL wearers with and without CL discomfort. Second, in this study, we did not investigate the effect on the viscosity and water retention of DQS LX and the electrostatic interactions among PVP, mucin and water. Further studies will need to be conducted to evaluate this issue. Third, we did not measure tear secretion volume at the baseline and after instillation of the eye drops. However, according to a previous study that investigated the tear fluid volume after instillation of DQS and artificial tear eye drops, these eye drops did not have an effect on the tear secretion volume [16]. Thus, it is our assumption that DQS LX also does not influence the tear secretion volume. Fourth, the present study design lacked a normal control, such as saline or artificial tears. In the future, we will need to investigate the effect of DQS LX as compared to a normal control. Moreover, it would have been better to enroll both eyes from the point of keeping the same　blink frequency related to tear turnover. In the present study, we used only the right eye because we were concerned that multiple measurements could potentially have an effect on the contralateral eye. This was especially the case for NIBUT with SCLs, when the measurements were conducted for more than 10 s, and thus the surface of the SCLs became hydrophobic and there are reports of cases in which the original condition was not recovered for at least a few seconds. Therefore, in the present study, only the right eye was enrolled, and a crossover design was utilized. Fifth, the subjective symptoms of SCL wearers are known to gradually increase towards the evening hours due to increasing friction between the SCLs and the eyelid or bulbar conjunctiva. Thus, at visits 2 and 3, measurements were performed after wearing SCLs for 7 h, as this study was attempting to investigate the effect of DQS LX on subjective symptoms encountered during real life. However, when SCLs are worn for 7 h, there is a significant increase in the tear component deposit [48]. Therefore, it is possible that we did not simply observe the relationship between the SCL surface properties and the parameters associated with tear film stability. As a result, our present findings may differ if we were only to consider a relatively short time period, such as after 15 min of wearing the SCLs.

In conclusion, DQS LX in SCL wearers was observed to have prolonged tear film stability, with increase in the amount of tear fluid, and relief in the subjective symptoms for longer periods than those observed for DQS. The present results suggest that instillation of DQS LX may be a countermeasure against dryness when wearing SCLs.
